# Official Policy Defined

**Published:** 1921-07-23

**Authors:** 


### THE VOLUNTARY HOSPITALS COMMISSION.

### Official Policy Defined.

The Minister of Health has issued a letter to the members of the Voluntary Hospitals Commission, whose names we published last week, p. 257, which embodies a clear definition of the Minister's policy and the terms of appointment of the Commission. We therefore publish it in full with the omission of the first (three) formal paragraphs. The cross-headings are inserted by us for the sake of clearness.

"The recommendations of the Cave Committee so far as Government grants are concerned were (1) that the State should provide a sum of £1,000,000 in aid of hospital deficits during the present year, and a further sum next year, and (2) that it should provide grants in aid of building extensions and improvements, pound for pound against contributions from other sources.

### No Money for Building Schemes.

"The Government have decided that they ought not to contribute towards building schemes when the hospitals are not paying their way and when the cost of building is steadily falling. They have further decided that in the present financial condition of the country they must be content to contribute £500,000 only in aid of deficits and rely on the public to provide the rest. Lord Cave's Committee recommended that a further grant should be made in 1922, but it should be clearly understood that the Government are unable to give further assistance than £500,000.

"The Government accept the view of the Cave Committee that the voluntary system must be maintained, and they have reluctantly assented to a special contribution from State funds because in the judgment of the Cave Committee the voluntary system would be gravely imperilled if the present temporary difficulties are not surmounted. But on the merits of the case they cannot admit that it is incumbent on the Government to find money to make up the present deficit on the voluntary hospitals, and they can only contemplate the provision of State assistance for a very limited period and simply and solely as a means of giving the voluntary hospitals a breathing space, during which they can re-establish their position, as the conditions resulting from the war disappear, and an opportunity of improving their organisation and developing their revenues.

### Community of Outlook and Co-ordinated Effort.

"Accordingly the essential duty of the Hospitals Commission is so to administer the Government grant as to secure that the necessity for such a grant shall disappear as quickly as possible. The development of fresh and permanent sources of revenue should be aimed at, as well as the securing of the utmost possible improvement in organisation and economy in administration compatible with the attainment of the great ends for which the hospitals exist. Controlling as it will a Government grant of half the estimated deficit, the Commission should be able to bring into the voluntary-hospital system a community of outlook and co-ordination of effort which it has been hitherto difficult to obtain, largely because the hospital system naturally embodies the defects as well as the merits of a voluntary system. The Commission will be in a position to render valuable aid both in supplying technical advice and experience on hospital management and economy, and also in advising as to fresh methods of obtaining voluntary assistance. They will no doubt examine the possibilities of special appeals from each hospital or group of hospitals or a general appeal throughout the country to make up, with the Government grant, a sufficient sum to meet the needs of the hospitals.

### Urgent Cases to be Helped First.

"They will consider, first of all, the case of those hospitals which have exhausted their realisable assets, and have been compelled to close wards which are urgently needed. In these exceptional cases such help may be given at once as is necessary to enable the hospital to maintain its normal complement of beds, grants being restricted within the limits of necessity, and full assurances being obtained that all reasonable steps have been, or are being, taken to reduce expense and raise new income. The information at my disposal does not enable me to say precisely how much money will be required to be spent in this way. The Commission will, of course, appreciate the fact that expenditure under this head must be restricted to absolute necessities since, in so far as it is incurred, it tends to make more difficult the securing of fresh money *pro rata* to the Government grant. When once the amount absolutely necessary is ascertained disbursement of the balance of the grant should proceed *pro rata* against fresh money raised or in sight up to a total of £500,000. The governing factor is that £500,000 is the limit of Government assistance, and is dependent on fresh money from other sources to an equivalent amount.

### Local Committees and their Functions.

"The Commission will be responsible for the appointment of local Voluntary Hospital Committees, following generally the recommendation on the subject in paragraphs 18-22 of the Report of Lord Cave's Committee. In the case of London the Commission will have the great advantage that there is already in existence the King Edward Hospital Fund, which will perform the functions of a local committee for the London area. The functions of the committees will be to act as local advisers to the Commission, to collect information as to the needs of their areas, to further co-operation between the hospitals in their areas, to co-ordinate appeals, and to assist in arranging transfer of patients where this can advantageously be done. They will also prepare, where practicable, schemes of co-operative purchase; they will organise systematic contributions by wage-earners in areas where no such system at present exists, and they will undertake the distribution of any contributions made by an Approved Society out of the valuation surplus in cases where the society is purely local in character.

"Applications for grants will be made in the first instance to the local Committee, and grants will be made by the Commission after consultation with them, though the Commission will not be bound to accept their recommendations. The primary functions of the Committees will be to stimulate and direct local effort; and it is of the greatest importance that the Commission should be able to secure the co-operation of men and women of good standing and local influence.

"The staff of the Commission will be provided by the Ministry. The administrative expenses of the local Voluntary Hospital Committees should be met from private sources, but in cases where local authorities are prepared to contribute sanction (where necessary) will be given under the Local Authorities (Expenses) Act."

